# The Associations among Psychological Distress, Coping Style, and Health Habits in Japanese Nursing Students: A Cross-Sectional Study

**DOI:** 10.3390/ijerph14111434

**Published:** 2017-11-22

**Authors:** Akio Tada

**Affiliations:** Department Faculty of Health Science, Hyogo University, 2301 Shinzaike Hiraoka-cho, Kakogawa, Hyogo 675-0195, Japan; atada@hyogo-dai.ac.jp; Tel.: +81-79-427-9908; Fax: +81-79-427-5112

**Keywords:** health habit, coping style, nursing student, psychological distress

## Abstract

**Background:** Nursing students in many countries have been reported to experience high levels of stress and psychological distress. Health habits could potentially mediate the association between coping styles and psychological status. The purpose of this study was to evaluate the mediation effect of health habits in the relationship between stress coping styles and psychological distress in Japanese nursing students. **Methods:** A total of 181 nursing students completed anonymous self-reported questionnaires comprised of the General Health Questionnaire-12 (GHQ-12), the Brief Coping Orientation questionnaire, and an additional questionnaire on health behavior. A mediation analysis using path analysis with bootstrapping was used for data analysis. **Results:** Multivariate linear regression analysis showed that psychological distress was significantly and positively associated with “Avoidance coping” (β = 0.39, *p* < 0.001), and was negatively associated with “Active coping” (β = −0.30, *p* < 0.001), “exercise habit” (β = −0.25, *p* = 0.001), and “sleeping” (β = −0.24, *p* = 0.002). In the path model, “Active coping” and “Avoidance coping” had significant or marginally significant associations with “exercise habits” (active: β = 0.19, *p* = 0.008, avoidance: β = −0.12, *p* = 0.088), and psychological distress (active: β = −0.25, *p* < 0.001, avoidance: β = 0.363, *p* < 0.001). However, these coping style variables did not have a significant association with “sleep”. In general, the size of the correlations was below 0.4. **Conclusions:** Exercise habits mediated the relationship between coping styles and psychological distress to a greater extent than sleep. The present study suggests the possibility that complex interactions between health habits and coping styles may influence the psychological status of nursing students.

## 1. Introduction

In many countries, nursing students, who have high workloads, have been reported as experiencing high levels of stress [1,2] and psychological distress [3,4]. Psychological distress is an important mental health issue that should be addressed in the field of nursing education since emotional disorders such as anxiety are considered to be components of psychological distress [5].

It has been reported that mental health is influenced by many factors, including coping styles and health behaviors. Appropriate coping strategies seem to contribute to a better mental health status [6,7]. Coping strategies have been suggested to mitigate the effect of stressors [8]. People with poorer health habits are more likely to have psychological distress [9,10,11,12]. Interventions promoting healthier lifestyles have been reported to improve mental status [13,14,15]. These studies have suggested that a healthy lifestyle reduces psychological stress.

Self-regulation is thought to contribute to health behavior and changes in health behaviors [16,17]. Coping styles have been found to be associated with personal self-regulation [18,19]. Since both health behavior and stress coping are involved in self-regulation, these factors are thought to be associated with each other. However, few studies have attempted to examine the link between health habits and coping styles [20,21]. Moreover, there is a possibility that health habits have an impact on the association between coping styles and psychological situations. However, the question of whether the associations between health habits and coping styles influence psychological status has not yet been studied. Understanding how health habits affect the association between coping styles and psychological situations may aid the efficient management of mental health. One possible hypothesis is that health habits function as an important variable in the association between coping styles and psychological status. In other words, health habits could potentially mediate the association between coping styles and psychological status.

This study aimed to evaluate the mediation effect of health habits in the relationship between stress coping styles and psychological distress in Japanese nursing students.

## 2. Methods

### 2.1. Study Design

A cross-sectional study design was utilized.

### 2.2. Participants and Data Collection

The subjects were 210 students registered in the Department of Nursing of Hyogo University, and included 106 second-year students and 104 third-year students. The students were asked to participate in the study after receiving an explanation of the study’s aims and procedures, the voluntary nature of the study, and the condition of anonymity at the beginning of two classes in compulsory subjects. After the explanation, the questionnaires were distributed. Only students who consented to participate in the study completed the questionnaires (101 second-year students and 99 third-year students). After a response period of 30 min, the students submitted the questionnaires by placing them in anonymous envelopes. The data were gathered in July and September of 2014.

### 2.3. Content of the Questionnaires

The questionnaires contained items regarding demographic factors (sex, year of study, living with family, and participation in club activities), the 12-item General Health Questionnaire (GHQ-12), the Brief Coping Orientation questionnaire, and a questionnaire on health behaviors.

#### 2.3.1. The 12-Item General Health Questionnaire

Distress levels were measured using the Japanese version of the GHQ-12, which is a widely used standard, composite measure of psychological distress [22] applicable to adolescents as well as adults. The validity and reliability of the Japanese version of the GHQ-12 have been confirmed (Cronbach’s α ≥ 0.80 for both sexes) [23]. The possible responses in the questionnaire are described as follows: “much less than usual”, “same as usual”, “more than usual”, and “much more than usual”. The standard scoring method recommended by Goldberg for the purpose of case identification is called the “GHQ method,” which is also known as the binary method. In this method the two least symptomatic answers are scored as 0 and the two most symptomatic answers are scored as 1 (i.e., 0-0-1-1). The minimum GHQ-12 total score is 0 and the maximum GHQ-12 total score is 12. The Cronbach’s alpha coefficient was 0.843 for this study.

#### 2.3.2. Brief Coping Orientation Questionnaire

The coping styles of the participants were assessed using the Japanese version of the Brief Coping Orientation (Brief COPE) questionnaire, a 28-item questionnaire that measures 14 dimensions of coping (self-distraction, active coping, denial, substance use, emotional, instruments, disengagement, venting, reframing, blaming, humor, acceptance, religion, and self-blaming) [24]. Each item was rated using a 4-point Likert scale as follows: “I haven’t been doing this at all” (1); “I’ve been doing this a little bit” (2); “I've been doing this a moderate amount” (3); and “I've been doing this a lot” (4). The Brief COPE [25] has been validated and is one of the most frequently used self-reported measures of coping responses. The Cronbach’s alpha coefficient was 0.734 for this study.

#### 2.3.3. Health Habits

Two health habits subscales were examined: the “exercise habit” subscale consisting of 4 items, and the “sleep” subscale consisting of 3 items. The subscale items were rated using a 5-point Likert scale ranging from “always” (1); “often” (2); “sometimes” (3); “rarely” (4); and “never” (5) or using a reverse Likert scale as follows: “never” (1); “rarely” (2); “sometimes” (3); “often” (4); and “always” (5). Consequently, better health habits received higher scores. The possible total scores for “exercise habits” ranged from 4 to 20, while those for sleep ranged from 3 to 15. The Cronbach’s alpha was 0.76 for the exercise habit subscale, and 0.72 for sleep subscale, indicating a high degree of internal consistency.

### 2.4. Ethical Considerations

This study was approved by the Hyogo University Ethics Committee (No. 14002).

### 2.5. Data Analysis

The statistical analyses were performed using SPSS Ver. 23 (IBM Corp, Armonk, NY, USA) and AMOS Ver. 24. (IBM Corp, Armonk, NY, USA) Differences of *p* < 0.05 were considered statistically significant. A Pearson correlation analysis was used to assess the inter-relationships among continuous variables. Multiple linear regression analyses were conducted to investigate factors associated with psychological distress. In the multiple linear regression analysis, each association was controlled for confounding factors (sex, year of study, living with family, and participation in club activities).

The structural equation model for the path analysis was constructed to analyze the direct and indirect effects of health habits on psychological distress. A model was established using coping styles as the independent variable, psychological distress as the dependent variable, and health habits as the mediators.

## 3. Results

One hundred and eighty-five students (92.5%) agreed to participate in the study and submitted completed questionnaires. Questionnaires missing data were excluded. Overall, 181 students completed the survey (90.5%).

### 3.1. Descriptive Analysis

The participants were predominantly female (89.5%). The numbers of second-year and third-year students were almost the same and together composed 97% of the population. Approximately 80% of the participants lived with their families. Most of the participants (63.0%) did not participate in club activities.

One hundred and eleven (61.3%) of the 181 participants had a GHQ-12 score ≥4, with a mean GHQ-12 score of 5.5 ± 1.1. The three most common coping styles adopted by the participants were acceptance (score 5.7), self-distraction (5.5) and planning (5.4). The least common coping styles were substance use, religion, and denial. The score for “exercise habits” was 9.7 (maximum score 20) while that for “sleep” was 8.1 (maximum score 15).

### 3.2. Correlations Between Variables

Table 1 shows the correlations between coping style items, health habit scales, and the GFQ-12 score. The Pearson r correlation coefficient showed that the GHQ-12 score had significant positive correlations with three Brief COPE items (disengagement: *r* = 0.25, *p* = 0.01; venting: *r* = 0.16, *p* = 0.036; and self-blame: *r* = 0.36, *p* < 0.001) and significant negative correlations with three Brief COPE items (active coping: *r* = −0.19, *p* = 0.009; reframing: *r* = −0.29, *p* < 0.001; and humor: *r* = −0.15, *p* = 0.045) and all the health habits that were examined (exercise: *r* = −0.26, *p* < 0.001; and sleeping: *r* = −0.19, *p* = 0.009). The correlations between coping style items and the health habit scales, and the GFQ-12 scores were below 0.4. The correlations between the items of the Brief COPE ranged from 0.02 to 0.72 and from −0.01 to −0.36.

To analyze how health behaviors mediated the relationships between psychological distress and the coping strategies that were significantly associated with psychological distress, “disengagement”, “venting” and “self-blame” were designated as “Avoidance coping”. “Active coping” included “reframing” and “humor” and “active coping”. The scores of these two subscales were equal to the sum of applicable coping items. The GHQ-12 score was significantly correlated with both “Active coping” (*r* = −0.28, *p* < 0.001) and “Avoidance coping” (*r* = 0.39, *p* < 0.001) in addition to health habits (Table 2). Exercise was significantly and positively correlated with “Active coping” (*r* = 0.20, *p* < 0.001) and tended to be significantly correlated with “Avoidance coping” (*r* = −0.13, *p* = 0.079) but sleeping did not exhibit such correlations (Table 2). In general, the sizes of the correlations were below 0.4.

### 3.3. Associations between Health Habits, Coping Styles, and Psychological Distress

To determine whether these significant correlations continued to exist after controlling for confounding factors (sex, year of study, living with family, and participation in club activities), a multivariate linear regression analysis was conducted. As a result, all the correlations were found to be significant after controlling for confounding factors. The GHQ-12 score was significantly and positively associated with “Avoidance coping” (β = 0.39, *p* < 0.001), and was negatively associated with “Active coping” (β = −0.30, *p* < 0.001), “exercise habit” (β = −0.25, *p* = 0.001) and “sleeping” (β = −0.24, *p* = 0.002).

### 3.4. Associations among Health Habits, Coping Styles, and Psychological Distress

**Figure 1:**
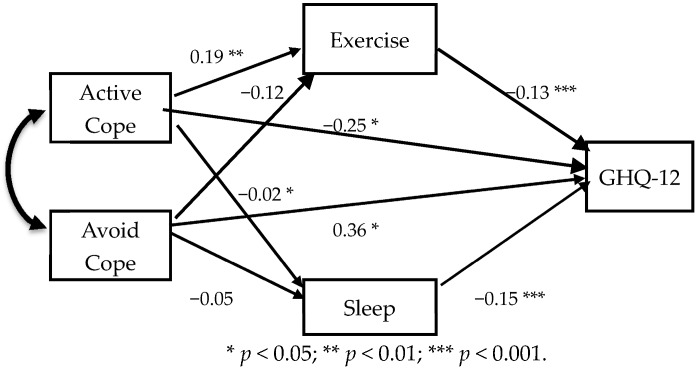
Model of mediation path analysis with coping styles for the effect between health habits and psychological distress.

The interrelationships among health habits, coping styles, and psychological distress were further investigated at the multivariate level using path analysis (Figure 1). “Active coping” had significant associations with “exercise habits” (β = 0.19, *p* = 0.008), and psychological distress (β = −0.25, *p* < 0.001). “Avoidance coping” had a marginally significant association with “exercise habits” (β = −0.12, *p* = 0.088) and a significant association with psychological distress (β = 0.363, *p* < 0.001). “Active coping” and “Avoidance coping” did not have a significant association with “sleep”. Psychological distress was associated with “exercise habit” (β = −0.14, *p* = 0.047), and “sleep” (β = −0.15, *p* = 0.024) as well as with health habits.

Table 3 summarizes the estimates of standardized direct, indirect and total effects of “Active coping” and “Avoidance” on psychological distress. The total effect (β = −0.27) of “Active coping” on psychological distress was comprised of a direct effect (β = −0.25) and an indirect effect (β = −0.02). “Avoidance coping” had a direct effect (β = 0.36) and an indirect effect (β = 0.02) on psychological distress (total β = 0.38). This path model yielded a good fit for exercise, self-blaming, and disengagement among the subjects (χ^2^ = 0.03, *p* = 0.847, GFI = 0.99, AGFI = 0.99, RMSEA < 0.001).

## 4. Discussion

The mean GHQ-12 score of the present subjects (5.5) was similar to that in a previously published study examining Japanese nursing students [26]. The most common and least common coping styles of the presently examined population were also similar to those reported in that study [26]. Because both populations were Japanese nursing students, the similarities in the GHQ-12 scores and coping styles between these studies are reasonable. Thus, the present subjects may not deviate from common Japanese nursing students.

The GHQ-12 score showed significant, positive associations with “Avoidance coping” (“disengagement”, “venting” and “self-blame”) and significant negative associations with “Active coping” (“reframing”, “humor”, and “active coping”). These coping styles have been reported to be associated with psychological disorders in many studies [27,28,29,30,31,32], consistent with the present results. The findings of this study contribute to revealing some of mechanisms by which coping styles have an influence on psychological distress. Similarly, the GHQ-12 score showed significant, positive associations with “sleep” and “exercise habits”. These results were also as expected because there is a large amount of evidence to support these associations [26,33,34,35,36,37] in cross-sectional studies. Furthermore, exercise reduced psychological distress in longitudinal studies [38,39,40]. It needs to be noted though that the strength of associations between psychological stress, coping styles, and health habits was relatively small in the present study, taken together, the findings in the present study suggested that the promotion of regular exercise and better sleep habits may be the most effective means of reducing psychological distress in the present study population.

Our study was the first to examine health habits as mediators in the relationship between coping styles and psychological distress in a sample of nursing students. It has been expected that individuals with better health habits tend to adopt “Active coping” rather than “Avoidance coping”. Furthermore, health habits may function as mediators between coping styles and psychological distress. Thus, individuals with better health habits who exhibit “Active coping”, rather than “Avoidance coping” are considered to be highly conscious of stress management. The results of the present study indicate that exercise habits have a considerable impact on psychological distress. Furthermore, exercise habits were significantly and positively associated with “Active coping” and were negatively associated with “Avoidance coping”, possibly leading to a lower level of psychological distress. Exercise may have an indirect influence via coping styles, in addition to a direct influence, on mental health. A diagram in which “exercise habits” have the potential to change the levels of psychological distress when mediated by coping styles can be hypothesized. Exercise was reported to be used as a coping strategy in the general population [41,42]. Subjects with “Active coping” might use exercise as a coping strategy. Those with “avoidance coping” were less likely to have “exercise habits”.

In contrast, the mediation effects of sleep in the relationship between coping styles and psychological distress were weak. Sleep appeared to be less effective than exercise for promoting “Active coping” and limiting “Avoidance coping”. The different associations with coping styles between exercise and sleep suggest that these health habits have different mechanisms for reducing psychological distress. Exercise increases levels of opioids such as β-endorphin [43,44,45], which produces a feeling of euphoria, dopamine [46], which plays a major role in reward-motivated behavior, and serotonin [47], which is thought to be a contributor to feelings of well-being and happiness. The mood elevation induced by opioids may be related to the adoption of “active coping”, rather than the evasion of “Avoidance coping”. Meanwhile, sleep is a mental and physical rest activity that is essential for human beings. The quality of sleep behavior is thought to prevent psychological distress regardless of coping styles. In other words, better sleep behavior, irrespective of coping styles, increases the possibility of a positive influence on mental health. However, the results obtained in this study might not always be observed in other populations. The association may vary depending on the characteristics of the population, such as age and ethnicity.

The present study found that the relationship between coping styles and psychological distress might be explained in part by exercise, particularly for “Active coping”. It appears that individuals who use more “Active coping” may have more exercise habits, which in turn reduce psychological distress to a greater degree. Since “Avoidance coping” is associated with psychological distress, the recommendation of having exercise habits may be effective for students with “Avoidance coping”. Mediation effects from other health habits with respect to coping styles and psychological distress are also expected. The elucidation of the mediation effects may help for the development of tools to improve mental health. Erdem et al. reported that health-related behaviors mediated the relationship between ethnicity and mental health [48]. Health habits may function as a mediator between mental health and mental health-related factors. Further investigation is necessary to understand the complexity of the relationship between health habits and coping styles and the influence of these factors on mental status.

### Limitations of Research

Several limitations of this study should be considered. First, the study results were based on self-reported data, which could represent a considerable overestimation or underestimation of coping styles or health habits.

Second, the design of this study used a cross-sectional survey, which cannot be used to infer causal relationships. Accordingly, longitudinal studies, particularly intervention studies with follow-up periods enabling changes in mental status, coping style, and health habits to be explored, would be beneficial.

Third, it is difficult to explain the development of psychological distress only by health behaviors and coping strategies because of the small size of correlation with psychological distress.

Further limitations of the present study include the relatively small sample size, and the small numbers of subjects in some subgroups. Analyses with a small sample size carry a risk of overestimating the impact of relationships between items evaluated statistically. In contrast, some significant associations might have been overlooked because the sample size was not powerful enough to detect them.

## 5. Conclusions

The present study highlighted the complicated association among psychological distress, coping style, and health behaviors in nursing students. Coping styles (“Avoidance coping”: “disengagement”, “venting”, and “self-blame”, and “active coping”: “Active coping”, “reframing” and “humor”) and health behaviors (“exercise habits” and “sleep”) were found to be significantly associated with psychological distress. Exercise habits may mediate the relationship between coping styles and psychological distress, but sleep may not. These findings provide beneficial information that could be used to improve the mental health of nursing students. Further studies addressing coping skill training or exercise habit promotion with individuals who have experienced psychological distress could provide beneficial information for mental health.

## Figures and Tables

**Table:** (**a**)

	GHQ-12	Exe	Sle	Dis	Act	Den	Sub	Emo	Ins
GHQ-12	※	−0.26 ***	−0.19 **	0.09	−0.19 **	0.06	0.07	−0.08	−0.01
Exe		※	0.28 **	−0.12	0.10	−0.03	0.02	0.04	0.12
Sle			※	−0.13	−0.01	−0.06	−0.13	0.06	−0.05
Dis				※	0.22 **	0.15 *	0.10	0.22 **	0.21 **
Act					※	−0.03	0.13	0.32 ***	0.41 ***
Den						※	0.36 ***	0.09	0.02
Sub							※	0.16 *	0.07
Emo								※	0.72 **
Ins									※
Dise									
Ven									
Ref									
Pla									
Hum									
Acc									
Rel									
Bla									

*: *p* < 0.05, **: *p* < 0.01, ***: *p* < 0.001; Exe: exercise habit, Sle: sleep, Dis: self-distraction, Act: active coping, Den: denial, Sub: substance use, Emo: use of emotional support, Ins: use of instrumental support, Dise: behavioral disengagement, Ven: venting, Pla: planning, Hum: humor, Acc: acceptance, Rel: religion, Bla: self-blame; GHQ-12: 12-item General Health Questionnaire.

**Table:** (**b**)

	Dise	Ven	Ref	Pla	Hum	Acc	Rel	Bla
GHQ	0.25 **	0.16 *	−0.29 **	−0.12	−0.16	−0.03	−0.02	0.36 ***
Exe	−0.12	0.01	0.18*	0.03	0.15 *	−0.03	0.03	−0.15 *
Sle	−0.04	0.05	−0.08	0.11	−0.02	−0.02	0.09	−0.06
Dis	0.26 **	0.24 **	0.14	−0.01	0.08	0.06	0.09	0.17 *
Act	−0.26 ***	0.13	0.39 ***	0.53 ***	0.24 **	0.42 ***	0.15 *	−0.52
Den	0.39 ***	0.07	0.06	−0.05	0.11	−0.31 ***	0.30 ***	0.18 *
Sub	0.04	0.09	0.07	0.02	0.15 *	0.01	0.23 **	0.04
Emo	−0.06	0.32 ***	0.28 ***	0.12	0.16 *	0.07	0.21 **	0.08
Ins	−0.13	0.33 ***	0.25 **	0.29 ***	0.06	0.28 ***	0.16 *	0.13
Dise	※	0.08	−0.14	−0.31 ***	0.08	−0.36 ***	−0.08	0.21 **
Ven		※	0.07	0.05	0.11	0.03	0.23 **	0.17 *
Ref			※	0.36 ***	0.34 ***	0.19 *	0.21 **	−0.05
Pla				※	0.09	0.49 ***	0.09	0.03
Hum					※	0.07	0.19 *	−0.12
Acc						※	−0.06	0.07
Rel							※	0.18 *
Bla								※

*: *p* < 0.05, **: *p* < 0.01, ***: *p* < 0.001; Exe: exercise habit, Sle: sleep, Dis: self-distraction, Act: active coping, Den: denial, Sub: substance use, Emo: use of emotional support, Ins: use of instrumental support, Dise: behavioral disengagement, Ven: venting, Pla: planning, Hum: humor, Acc: acceptance, Rel: religion, Bla: self-blame.

**Table 2:** Associations between psychological distress, health habits and coping styles (Active coping and Avoidance coping).

	GHQ-12	Exercise	Sleep	Active coping	Avoidance coping
GHQ-12	※	−0.26 ***	−0.19 **	−0.28 ***	0.39 ***
Exercise		※	0.28 ***	0.20 **	−0.13
Sleep			※	0.17 *	−0.05
Active coping				※	−0.05
Avoidance coping					※

*: *p* < 0.05, **: *p* < 0.01, ***: *p* < 0.001.

**Table 3:** Summary of the direct, indirect, and total effects of significant factors on psychological distress among Japanese nursing students.

Variables	Effects	Significance Factors			
		Exercise	Sleep	Active Coping	Avoidance Coping
Psychological	Direct	−0.13	−0.15	−0.25	0.36
distress	Indirect	0.00	0.00	−0.02	0.02
	Total	−0.13	−0.15	−0.27	0.38
